# Safety and Feasibility of Lin- Cells Administration to ALS Patients: A Novel View on Humoral Factors and miRNA Profiles

**DOI:** 10.3390/ijms19051312

**Published:** 2018-04-27

**Authors:** Anna Sobuś, Bartłomiej Baumert, Zofia Litwińska, Monika Gołąb-Janowska, Jacek Stępniewski, Maciej Kotowski, Ewa Pius-Sadowska, Miłosz P. Kawa, Dorota Gródecka-Szwajkiewicz, Jarosław Peregud-Pogorzelski, Józef Dulak, Przemysław Nowacki, Bogusław Machaliński

**Affiliations:** 1 Department of General Pathology, Pomeranian Medical University, 70-111 Szczecin, Poland; ania.sobus@gmail.com (A.S.); baumertbartlomiej@gmail.com (B.B.); z.litwinska@gmail.com (Z.L.); maciej.j.kotowski@gmail.com (M.K.); ewapius@wp.pl (E.P.-S.); kawamilosz@gmail.com (M.P.K.); d.szwajkiewicz@op.pl (D.G.-S.); 2 Department of Neurology, Pomeranian Medical University, 71-252 Szczecin, Poland; monikagj@op.pl (M.G.-J.); kneurolo@pum.edu.pl (P.N.); 3 Department of Medical Biotechnology, Faculty of Biochemistry, Biophysics and Biotechnology, 30-387 Krakow, Poland; jacek.stepniewski@uj.edu.pl (J.S.); jozef.dulak@uj.edu.pl (J.D.); 4 Department of Paediatric Oncology, Pomeranian Medical University, 71-252 Szczecin, Poland; peregud@tstd.pl

**Keywords:** amyotrophic lateral sclerosis (ALS), Lin- cells, neurotrophins, complement, microRNA, trophic effects of stem cell-based therapy

## Abstract

Therapeutic options for amyotrophic lateral sclerosis (ALS) are still limited. Great hopes, however, are placed in growth factors that show neuroprotective abilities (e.g., nerve growth factor (NGF), brain-derived neurotrophic factor (BDNF), and vascular endothelial growth factor (VEGF)) and in the immune modulating features, in particular, the anti-inflammatory effects. In our study we aimed to investigate whether a bone marrow-derived lineage-negative (Lin-) cells population, after autologous application into cerebrospinal fluid (CSF), is able to produce noticeable concentrations of trophic factors and inflammatory-related proteins and thus influence the clinical course of ALS. To our knowledge, the evaluation of Lin- cells transplantation for ALS treatment has not been previously reported. Early hematopoietic Lin- cells were isolated from twelve ALS patients’ bone marrow, and later, the suspension of cells was administered into the subarachnoid space by lumbar puncture. Concentrations of selected proteins in the CSF and plasma were quantified by multiplex fluorescent bead-based immunoassays at different timepoints post-transplantation. We also chose microRNAs (miRNAs) related to muscle biology (miRNA-1, miRNA-133a, and miRNA-206) and angiogenesis and inflammation (miRNA-155 and miRNA-378) and tested, for the first time, their expression profiles in the CSF and plasma of ALS patients after Lin- cells transplantation. The injection of bone marrow cells resulted in decreased concentration of selected inflammatory proteins (C3) after Lin- cells injection, particularly in patients who had a better clinical outcome. Moreover, several analyzed miRNAs have changed expression levels in the CSF and plasma of ALS patients subsequent to Lin- cells administration. Interestingly, the expression of miR-206 increased in ALS patients, while miR-378 decreased both in the CSF and plasma one month after the cells’ injection. We propose that autologous lineage-negative early hematopoietic cells injected intrathecally may be a safe and feasible source of material for transplantations to the central nervous system (CNS) environment aimed at anti-inflammatory support provision for ALS adjuvant treatment strategies. Further research is needed to evaluate whether the observed effects could significantly influence the ALS progression.

## 1. Introduction

Amyotrophic lateral sclerosis (ALS) is a progressive neurodegenerative disease that selectively affects motor neurons. The typical symptomatic hallmark involves muscle weakness and wasting, cramps, poor reflexes, twitching, and speech problems. The worldwide incidence is estimated to be 2–3 per 100,000, and most patients have first symptoms in their sixth and seventh decades [1]. The majority of patients diagnosed with ALS are classified as having sporadic disease, whereas 5–10% have a positive family history [2]. To date, several genes and loci of major effect have been identified, and among the known genes, mutations in SOD1 (superoxide dismutase 1) account for 20% of familial ALS [1].

During the neurodegeneration of ALS, dysfunction occurs at both the motor neuron cell body and peripheral motor axon. The postulated pathophysiological mechanisms responsible for motor neurons degeneration involve oxidative stress, excitotoxicity, mitochondrial dysfunction, and protein aggregates abnormally activating major histocompatibility complex class I [3,4,5]. Recently, it was shown that neuroinflammation mediated by glial cells and systemic immune activation may play a key role in the progression of the disease, through mechanisms that can be either neuroprotective or neurodetrimental, depending on the type of cells and the motor neuron compartment involved [6]. Several studies have indicated that the immune system is actively involved in the progression of ALS—CNS, microglia and astrocytes are activated during disease progression, whereas peripheral T lymphocytes and natural killer cells infiltrate the spinal cord. Previous studies have shown that many proteins contributing to inflammation and/or the innate immune system may exhibit high levels in the CSF, plasma, or tissues of ALS patients (e.g., tumor necrosis factor alpha (TNF-α), circulating chemokines, and cytokines). Among them, toll-like receptors (TLRs) have an important part in the formation of chronic inflammation in neurodegenerative illnesses such as ALS [7]. It has also been stated that ALS patients present signs of systemic inflammation, reflected in increased levels of C-reactive protein (CRP) and complement components, such as C3 and C4 [8,9].

The currently available treatment option for ALS in Poland is riluzole, which prolongs life by 2–3 months with debatable functional improvement [10]. Although the Food and Drug Administration (FDA) has recently approved the use of edaravone, a free radical scavenger, at this moment, it is not yet registered in Poland [11]. The broad spectrum of molecular and cellular players in ALS certainly makes drug development difficult. Recently, increased emphasis has arisen on novel treatment options for neurodegenerative disorders using stem cell-based therapies, which have been widely investigated in two major directions: cell replacement and trophic support. The first approach aims at replacing the degenerated neurons, while the latter claims that stem/progenitor cells (SCs/SPCs, respectively) might provide a neuroprotective microenvironment for damaged tissue and save the remaining neurons [12]. In recent years, numerous studies have shown that stem cell transplantation elicits neurogenesis and angiogenesis as stem cells release numerous growth factors, which regulate the growth, differentiation and migration of neural SPCs [13,14]. It seems that in ALS, in the absence of one specific causative mechanism identified, the protection of the motoneurons with multipotent growth factors remains the most rational approach available. So far, the majority of studies and clinical trials in ALS have been focused on neurotrophic factors, but also other growth factors have been investigated for their not-so-obvious neurotrophic properties (e.g., vascular endothelial growth factor (VEGF)).

Three distinct families of neurotrophic factors (NTFs) have been characterized in recent years: classical neurotrophins (e.g., nerve growth factor (NGF), brain-derived neurotrophic factor (BDNF), neurotrophin 3 (NT-3), and neurotrophin 4 (NT-4)); the glial cell line-derived neurotrophic factor family of ligands (GDNF), neurturin (NRTN), artemin (ARTN), and persephin (PSPN)); and neuropoietic cytokines [15]. Neurotrophins (NTs) possess strong neuroprotective properties and determine neuronal homeostasis by inducing neural SPCs proliferation, migration, and differentiation via specific/nonspecific receptor interactions [16]. They are also responsible for the prevention of oxidative stress and apoptosis inhibition [17]. The accessibility of neurotrophic factors in optimal levels is critical during development, but they also regulate motor neuron maintenance and survival even long after neurons have become fully differentiated. NTs might be an interesting treatment tool for various neurodegenerative disorders; however, their poor pharmacokinetic profiles (primarily their inability to cross the blood–brain barrier (BBB) and short half-life) make their application in a protein form largely ineffective [18,19].

Recently, a great therapeutic potential is being attributed to vascular endothelial growth factor (VEGF), which, on the contrary to NTs, does cross the BBB in sufficient concentrations [20]. Besides its prominent role in angiogenesis, VEGF also exerts important protective effects on neurons. VEGF promotes the survival of cells overexpressing a mutated SOD1 G93A protein [21,22] and ameliorates the effects of glutamate excitotoxicity [23]. In rodents, VEGF treatment leads to a delay of ALS onset, an improvement of motor functions, the protection of motoneurons and neuromuscular junctions, and increase in survival [24,25]. Overall, pre-clinical data on the VEGF role in ALS treatment is promising.

Multiple mechanisms regulate proper levels of protein expression; among these, microRNAs (miRNAs, miRs) are commonly seen as ‘master regulators’ [26]. MiRNAs are small, non-coding molecules, which regulate gene expression at the post-transcriptional level, usually binding the 3′-untranslated region of mRNA sequence [27]. Apart from their recognized role in cellular specification and pathophysiological mechanisms, the recent discovery of circulating miRNAs also suggests that they provide novel means for paracrine and systemic communication [28]. In regards to ALS, recent studies have shown a correlation between miRNAs expression and microglia activation [29]. A group of muscle-specific miRNAs (“myo-miRs”) including miRNA-1, miRNA-206, and miRNA-133a/b, which are involved in the process of proliferation, plasticity, regeneration, and repair of muscle [30], might play a role in modulating the disease course in ALS, where degeneration of both upper and lower motoneurons leads to muscle atrophy. Recent findings also demonstrate a presumable role of other groups of miRNAs, including immune system-related miRNAs (“immuno-miRs”), such as miRNA-155 [31,32], and angiogenic-related miRNAs (“angio-miRs”), such as miRNA-378 [33], in ALS pathology. In this study, we chose to investigate the expression levels of miR-1, -133a, -155, -206, and -378 for their role in the pathophysiological processes in ALS. To our knowledge, miRNA expression in the physiological fluids of ALS patients during the course of experimental lineage-negative (Lin-) cell therapy has not been studied so far.

We have previously shown that umbilical cord blood (UCB)-derived SPCs, especially Lin- cells, strongly and specifically express classical NTs and the novel neurotrophic cytokines: cerebral dopamine neurotrophic factor (CDNF), mesencephalic astrocyte-derived neurotrophic factor (MANF), pigment epithelium-derived factor (PEDF), and the aforementioned VEGF. We have also shown that these secreted factors support neuronal proliferation and in vitro survival in a conditioned medium from Lin-SPCs [14]. In this study, we aimed to investigate whether bone marrow-derived Lin- cells administration is safe and feasible in ALS patients. Because miRNAs and growth factors have a strong role in ALS pathology, we hypothesized that this adjuvant cell therapy could also bring specific changes in the expression profiles in both the CSF and blood plasma of ALS patients.

## 2. Results

### 2.1. Analysis of Included Individuals

Four females and eight males suffering from ALS were enrolled in the study. The demographic and neurological characteristics of the ALS patients are shown in Table 1. There were no abnormalities on the laboratory tests at the inclusion to the study. Clinical analysis was performed in 12 patients as described in Section 4. All ALS patients enrolled in this trial were treated once with autologous bone marrow-derived lineage-negative early hematopoietic cell transplantation given intrathecally to the spinal canal and were assessed for therapy safety and for analysis of selected growth factors, cytokines, and miRNA profiles expressed in the blood plasma and cerebral spinal fluid. In our previous studies, we have thoroughly characterized Lin- cells as a heterogeneous population, which consists of precursor, progenitor, and stem cells and lacks mature morphotic blood elements [15]. Of note, inter-individual variability related to the number of Lin- cells isolated from the bone marrow from each ALS patient and subsequently transplanted intrathecally underlines the heterogeneity of the study patients’ groups (Table 1). In three months post-transplantation, 12 patients were retrospectively analyzed in detail as described in Section 4. As a result of such investigation, the whole patient population was divided into two different groups based on the disease course post-transplantation. Group I included six patients who did not decline neurologically after Lin- cells transplantation. In contrast, group II consisted of six patients, who presented deterioration of neurological status despite cellular adjuvant therapy. Then, in the context of the ALS course, further analysis of molecular data was performed separately for both groups and compared together. Initial comparison of the anthropometric parameters between both groups of treated individuals revealed no statistically significant differences (Table 1). In our study, patients from group II appeared to obtain lower number of Lin- cells per transplantation than individuals from group I.

We have not observed any adverse effects of autologous bone marrow-derived Lin- cells injected intrathecally, which makes them a safe and feasible source of material for transplantations to the CNS environment aimed at trophic support provision. The characteristics of both groups are shown in Table 1. Laboratory tests were performed on the obtained peripheral blood samples, and the results are presented in the Supplementary Materials.

### 2.2. Growth Factor Levels in CSF

**Figure 1:**
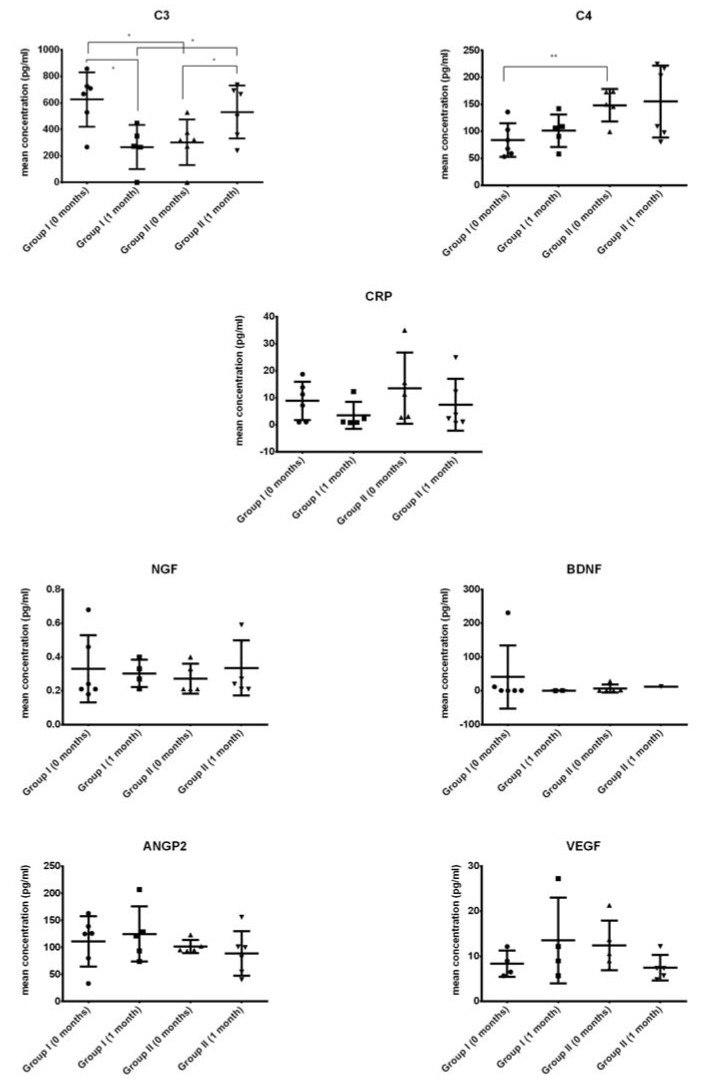
Levels of selected growth factors and cytokines in ALS patients’ CSF at different timepoints (0 months: before Lin- cells transplantation, 1 month: 1 month after Lin- cells transplantation) and their statistical comparison. A description of the difference between group I and II is given in Section 4. * *p* < 0.05, ** *p* < 0.01—level of significance. CRP: C-reactive protein; NGF: nerve growth factor; BDNF: brain-derived neurotrophic factor; ANGP2: angiopoietin 2; VEGF: vascular endothelial growth factor. Symbols (solid circle, triangle, square) are used to distinguish groups, each symbol represents one patient.

The concentrations of inflammatory proteins (C3, C4, and CRP), neurotrophic factors (NGF and BDNF), and growth factors involved in angiogenesis (ANGP2 and VEGF) were assessed (Figure 1).

We found that levels of C3 were significantly lower (up to two times) after Lin- cells transplantation in ALS patients from group I than before transplantation (Tx). In contrast, C3 concentration after Lin- cells transplantation in ALS patients from group II was significantly higher than before cell administration (Figure 1). Interestingly, when we compared the levels of C3 before cell therapy in patients diagnosed with ALS from both groups included in the study, we observed significantly higher (up to two times) C3 concentration in patients from group I. In addition, the analysis of the initial concentration of C4 before Lin- cells transplantation revealed a 50% increase in the individuals from group II. We observed no significant differences in the levels of neurotrophic (NGF and BDNF) and angiopoietic (ANGP2 and VEGF) growth factors between the individuals from both groups of patients diagnosed with ALS neither before nor after Lin- cells transplantation (Figure 1). However, we cannot compare the obtained data with the control group values due to the lack of a control group for the procedure in this preliminary study, which is explained in Section 3.

### 2.3. Comparison of Systemic Levels of Neurotrophic Growth Factors in Blood Plasma from ALS Patients

**Figure 2:**
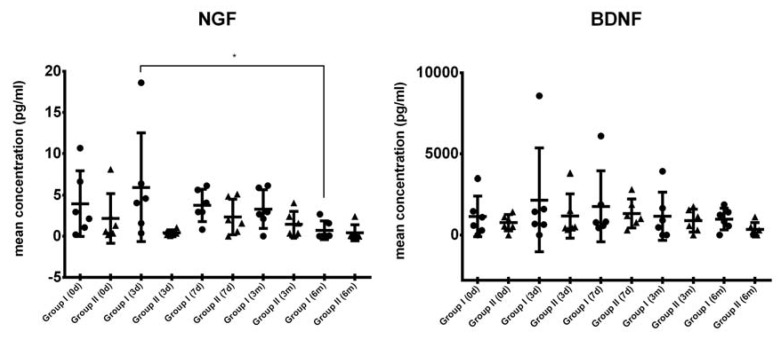
Levels of selected neurotrophic growth factors in ALS patients’ blood plasma at different timepoints (0, 3, and 7 days and 3 and 6 months after the experiment onset) and their statistical comparison. Description of the difference between group I and II is given in Section 4. * *p* < 0.05—level of significance. Symbols (solid circle, triangle, square) are used to distinguish groups, each symbol represents one patient.

Using multiplex immunoassay, we have also assessed the levels of NGF and BDNF in plasma samples (Figure 2). We have observed that in group I, NGF levels considerably increased in the first days after cells injection; however, they began to gradually decrease after a week, presenting with the lowest concentration in six months post-transplantation at the end of the investigation. Interestingly, in group II of ALS patients, the levels of NGF were considerably decreased in all study time-points compared with the corresponding time-points in group I before and after cell therapy. General tendency in the changes of concentration of BDNF in plasma were similar in both groups; although, in group I, we have noted an increase in BDNF level in the third day post-transplantation (from 1382.6 pg/mL to 2588.4 pg/mL). In group II, the upswing was much lower indeed (from 915.6 pg/mL to 1172.0 pg/mL). However, the only statistically significant difference was observed for NGF in group I (three days vs. six months), but the interpretation must be cautious due to the large variation in the levels of NGF, the small number of patients investigated, and the lack of a control group.

### 2.4. miRNA Expression

**Figure 3:**
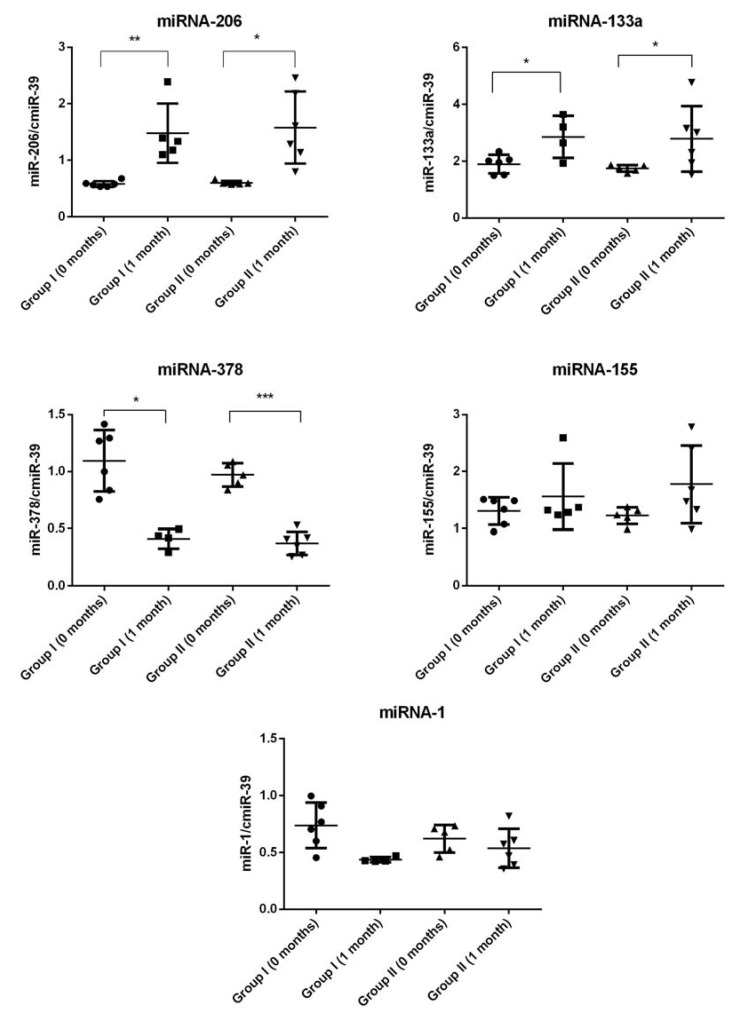
Mean relative values of microRNAs (miRNA) expression in ALS patients’ CSF (0 months: before Lin- cells transplantation, 1 month: 1 month after Lin- cells transplantation); * *p* < 0.05, ** *p* < 0.01, *** *p* < 0.001. Symbols (solid circle, triangle, square) are used to distinguish groups, each symbol represents one patient.

**Figure 4:**
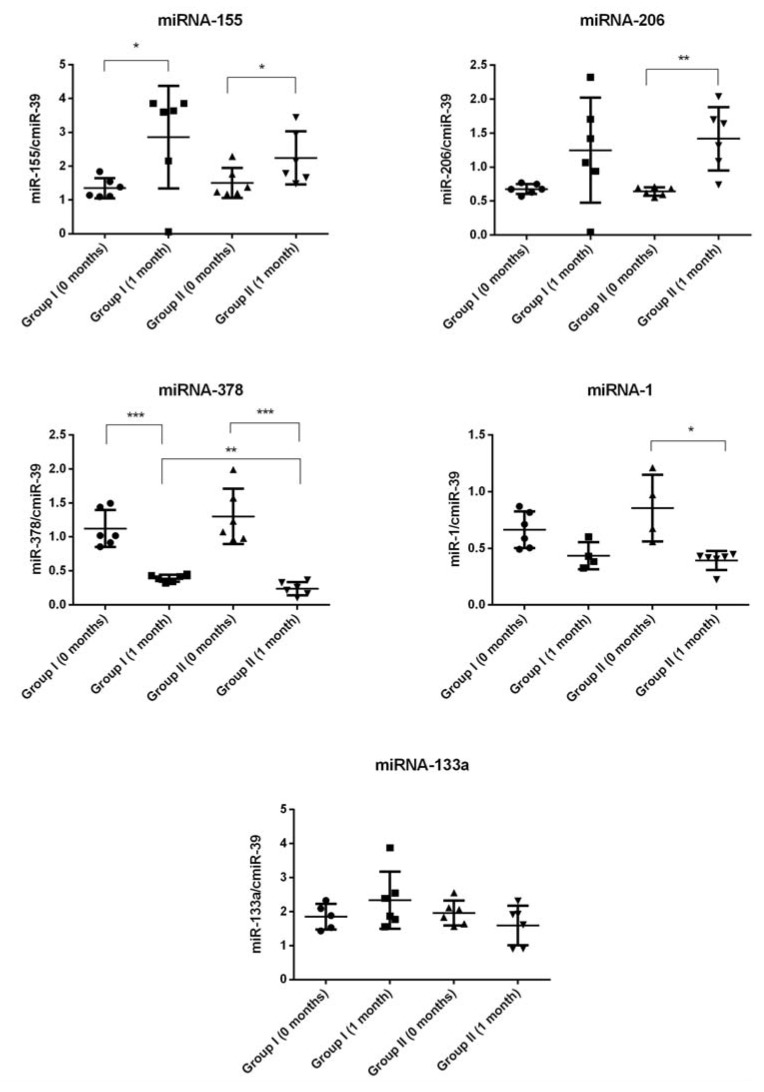
Mean relative values of miRNA expression in ALS patients’ blood plasma (0 months: before Lin- cells transplantation, 1 month: 1 month after Lin- cells transplantation); * *p* < 0.05, ** *p* < 0.01, *** *p* < 0.001. Symbols (solid circle, triangle, square) are used to distinguish groups, each symbol represents one patient.

Our results of miRNA expression analysis in the CSF and plasma are depicted in Figure 3 and Figure 4 respectively. We found that in the CSF, the expression levels of miRNA-206 were significantly higher (up to two times) in ALS patients from both groups after cell transplantation compared to the initial timepoint before Lin- cells administration. Interestingly, relatively similar significant results were observed in case of miRNA-133a in both groups of ALS patients before and after cell Tx. In contrast, we found that levels of miRNA-378 were significantly lower one month after Lin- cells therapy in both groups of ALS patients. We did not detect any difference between experimental timepoints when analyzing the expression pattern of miRNA-1 and miRNA-155 in the CSF.

Next, we became interested in systemic levels of selected miRNAs in the peripheral blood plasma. We found that in plasma, the expression levels of miRNA-155 were significantly higher (up to two times) in ALS patients from both groups after cell transplantation compared with the initial timepoint before Tx of Lin- cells. Furthermore, in group II of ALS patients, we detected a similar expression pattern in case of miRNA-206 before and after cell administration. In group I of ALS patients, miRNA-206 was also increased nearly 100% after Lin- cells Tx compared with the initial timepoint; however, in this case, the difference did not reach statistical significance. In contrast, we found that levels of miRNA-378 were significantly lower (maximally up to three times) one month after Lin- cells therapy in both groups of ALS patients. Likewise, Lin- cells administration induced a significant decrease of miRNA-1 expression in group II of ALS patients one month post-injection. We did not detect any difference between experimental timepoints when analyzing the expression pattern of miRNA-133a in plasma.

## 3. Discussion

ALS is an incurable neurodegenerative disease, and despite the substantial number of approaches that have been tested clinically, therapeutic options are still limited. Many hopes for the cure for ALS are placed in growth factors that show neuroprotective abilities and are known to promote the survival of neurons (e.g., NGF, BDNF, and others). However, their plasma delivery in pure protein form is rather ineffective, often due to their poor pharmacokinetic profiles and inability to cross the blood–brain barrier in substantial amounts [34]. Therefore, in ALS, different stem and progenitor cell therapy approaches step forward, as stem cells are avid growth factors ‘producers’. Human intrathecal transplantation of different stem cell populations, including bone marrow (BM)-derived hematopoietic stem and progenitor cells, have been previously attempted in patients with ALS, as well as in other neurological disorders, such as stroke, cerebral palsy, or injury of the spinal cord, with promising results and no relevant complications [35,36]. Importantly, in the experimental studies with stem cell therapies performed in ALS patients, different quantities of cells were transplanted, even in the same cohorts of subjects, due to the limited number of isolated cells available for a given patient, however, with good study outcomes [37]. In this study, we investigated the safety and feasibility of the administration of, previously described by our team [15], lineage-negative early hematopoietic progenitor cells derived from each patient’s own bone marrow and their influence on the clinical course of ALS through the first, to our knowledge, intrathecal transplantation of Lin- cells at different doses to ALS patients. This selectively isolated cell population shows long-term self-renewal and paracrine secretion of various angiopoietic and neurotrophic factors [14,38,39] on much higher levels than do other nucleated cells [15]. Therefore, we have focused on potential/suggested trophic activity exerted by transplanted Lin- cells in a neural environment rather than on their direct engraftment and replacement of degenerating neurons in the nervous system affected by ALS. We have also tested for the first time whether Lin- cells transplantation might influence specific miRNA expression patterns observed in the CSF and in the peripheral blood plasma of ALS patients.

The important role of neuroinflammation mediated by glial and immune cells that are active players in the pathogenesis of ALS is emerging [17]. Therefore, we have focused first on neuroinflammation in our ALS patient population, as numerous inflammatory proteins have been previously shown to be altered by this disease [40,41]. In our study, there were significant differences in C3 and C4 concentration between study groups. Importantly, before cell transplantation, group I presented higher C3 and lower C4 levels than group II. After Lin- cells transplantation, C3 levels decreased in group I, but increased in patients from group II, whose neurological condition deteriorated in course of ALS.

It was reported from the analysis of postmortem ALS spinal cord tissue that a robust complement activation has been detected at RNA and protein levels in ALS patients, leading to increased amounts of different activated complement components in the CSF and plasma, as well as directly in the spinal cord [42,43]. Sta et al., have recently suggested that there is disruption of the balance of clearance and repair in the CNS of ALS patients, which involves the complement system [44]. In a model of immune activation in ALS proposed by this group, the stressed neurons and the surrounding glial cells produced complement C1q and C4. C4 has been also shown to play a role in the attraction and activation of macrophages in SOD1 mutant mice [45]. C1q represents the initiating component of the classic complement pathway (C1→C4/C2/C3→C5) [46], whereas C3 is central to the classic and the alternative complement pathway (C3→C3/B→C5) [47]. Although most complement components are produced by the liver, many different cell types, including CNS microglia, specific neuronal populations, and astrocytes, can produce C3 and C4 [48]. Activation of the complement system and pro-inflammatory microglia may result in the impairment of the BBB; therefore, the increased levels of C3 and C4 in the CSF in ALS might result from both the increased intrathecal synthesis and the influx from the systemic circulation.

Several studies in ALS mice models have correlated the increase in complement components with the disease progression, which might in part also explain our findings. In a recent study, deletion of the gene encoding C4 did not affect the disease course in high-expressing mutant SOD1G93A mice [45]; however, the C3-dependent alternative complement pathway activation would still be possible. Local activation and increased expression of complement components signaling can contribute also to the recruitment of different mononuclear cells, including macrophages, that might accelerate the neuronal death, as observed in SOD1G93A mice [49]. In this notion, previously described selective inhibition of C5a–C5aR1 signaling ameliorated ALS pathology, reduced motor symptoms, and extended the survival of SOD1G93A mice [50]. Likewise, in our recent study on the constitutive C3-deficiency in the neural retina, it was found that the lack of activated C3 resulted in lower expression of the cellular oxidative stress components and higher anti-apoptotic activity together with significantly increased expression of several gene sets associated with the maintenance of the physiological functions of the nervous system [51]. Our actual observation of the significantly decreased C3 levels in the CSF in group I after Lin- cells administration may suggest temporary inhibition or at least a slowdown of neuroinflammatory processes, perhaps at least partially induced by Lin- cells transplantation. In contrast, the increase of C3 expression in group II of ALS patients might be a reflection of continuous local complement activation, which was not blocked sufficiently by the Lin- cells therapy approach. It should be stressed here that group II of ALS patients obtained a lower number of Lin- cells during transplantation in general compared to the ALS individuals representing group I in our study.

Neurotrophic factors have been at the center of interest in ALS research for years. Neurotrophins are proposed as potential drugs and have been used in multiple approaches to decrease the disease progression in human, as well as in animal models [52,53,54,55,56]. In our study, we have observed that a single injection of Lin- cells does not result in a prolonged change of BDNF and NGF levels in the CSF. In fact, due to the short half-life of both molecules, it might be the case that their concentrations in the CSF have changed shortly after cell injection, but in this study we examined the CSF collected before the Lin- cells transplantation and after one month, which could have been too distant a timepoint to observe actual changes in these proteins’ concentrations. We can expect that the potential beneficial effects of elevated neurotrophins concentrations in the CSF of ALS patients may possibly be prolonged with repeated cell injections, but this approach would require additional cell isolations and further monitoring timepoints. Nevertheless, a portion of NTs released by the administered Lin- cells might as well infiltrate from the CSF through the BBB and thus temporarily influence the local NTs synthesis within other cells, mostly in patients from group I, who received a greater number of Lin- cells. Interestingly, we previously observed that the exposure of Lin- cells to NTs, could increase the local expression of other NTs, such as NT-3 expression following BDNF exposure [57]. Therefore, the detected plasma concentration of BDNF and NGF might be a reflection of a systemic response after Lin- cells transplantation but could also arise from increased expression of NGF in skeletal muscle [58] or altered astrocyte–motoneuron cross-talk [59]. The observed results might also be due to generally low, often undetectable, levels of these proteins in the blood plasma [60], which make NTs concentration assessment challenging. This hypothesis is supported by the fact that transcripts for BDNF are strongly downregulated in muscle biopsies from ALS patients [61]; thus, the NTs released from Lin- cells may just cover the basic demands for these regulatory proteins in the nervous system and other tissues of ALS-affected human organisms.

MiRNAs operating as fine tuners of post-transcriptional events mediate neuronal gene expression, and their dysregulation becomes instrumental for understanding many diseases [62,63], including ALS. ALS is a motor neuron disorder, and it has been proven in various reports that ALS can be caused due to dysregulation of miRNAs and thus misexpression of proteins in cells. Recently, it has been postulated that disrupted signaling at the neuromuscular junction, caused by cytotoxicity associated with abnormal glutamate clearance and an overactive inflammatory response, results in neuromuscular degeneration. Dysregulation of key miRNAs triggers the alterations in motor neuron physiology, resulting in ALS pathology. Therefore, in the present study, we tested the expression profiles of five chosen miRNAs (myo-miRs, angio-miRs, and immuno-miRs) in the plasma and CSF of patients at two timepoints: before Lin- cells transplantation and a month after. Previous studies have also stressed that microRNA profiling in ALS patients is largely dependent on the disease duration and the patient’s sex and age at onset [64], and all these factors might have influenced our results. Overall, we have observed similarities in miRNA profiles between groups I and II. Because the number of Lin- cells transplanted varied between the groups, our miRNA results may suggest that the number of transplanted cells is not a main factor influencing miRNA profiles. However, the significant changes of several miRNA expression levels after Lin- cells administration may represent subtle neurological mechanisms modifications induced by the presence of biologically active Lin- cells in the area of the central nervous system after their intrathecal transplantation. Nevertheless, several miRNAs investigated in this study could potentially serve as biomarkers of disease activity and confirm previous observations in this field of molecular neurology.

In the group of myo-miRs, the key regulator of the signaling between the motor neurons and skeletal muscle fibers at the neuromuscular synapses is miR-206—a skeletal muscle-specific microRNA affected in course of ALS. It has been found that deficiency of miR-206 in the ALS mouse model (SOD1G93A mice) accelerates disease progression, as miR-206 is required for efficient regeneration of neuromuscular synapses after nerve injury, which process probably accounts for the salutary effects in ALS. Of note, miR-206 mediates these effects through histone deacetylase 4 and fibroblast growth factor signaling pathways in ALS-affected muscle cells [65]. In our study, the expression levels of this molecule were altered in all ALS patients recruited to this study, and we observed a significant increase in miR-206 levels one month after Lin- cells transplantation among both groups of ALS patients (groups I and II). Importantly, a similar pattern of expression changes in both study groups was observed after Lin- cells transplantation and also in case of another myo-miR—miR133a. Increased expression levels of both analyzed myo-miRs post-transplantation could have positive regulatory effects by promoting the formation of new synapses that may delay the progression of ALS in our patient cohort after Lin- cells transplantation. This observation is in concordance with the findings of previous studies that muscles upregulate these miRs in a futile attempt to promote reinnervation after their damage and thus resist disease progression [65].

Neuroinflammation and glial cell activation have been shown to contribute to motor neuron degeneration in ALS. The upregulation of “inflamma-miRNAs”, also known as “immune-miRs”, including miR-155, was shown to contribute to microglia-mediated immune response and neuroinflammation [66]. Thus, after Lin- cells administration we defined alterations of miR-155 expression, noticeably important for neuroinflammation-associated signaling pathways in ALS. Interestingly, we found that the significant increase of miR-155 expression levels was a sustained feature only in the blood plasma collected from all ALS patients after Lin- cells transplantation but not in their CSF samples. The constant expression profile of miR-155 in the CSF after cell transplantation might vaguely suggest that the inflammation is not progressing [67] after cell transplantation. On the other hand, there is recent evidence demonstrating that upregulation of miR-155 benefits in preventing the elevation of other pro-inflammatory cytokines. The miR-155 upregulation could be due to the lack of CX3CL1 inhibitory input from neurons, essential for the microglia surveilling phenotype [68]. In addition, the protective and anti-inflammatory role of miR-155 was recently suggested in the model of atherosclerosis-associated chronic inflammation [69]. Therefore, the results of miR-155 expression in our study may provide a potential explanation for the observed significantly lower concentration of pro-inflammatory C3 molecule in the CSF from ALS patients after Lin- cells transplantation.

MiR-378 is strongly implied in adipocyte gene expression and lipogenesis [70]. Taken above, miR-378 might regulate adiponectin expression through a C/EBP binding site of the adiponectin promoter [70]. MiR-378 is significantly upregulated in muscle cachexia, which can gradually develop in patients affected by ALS. The expression of miR-378 associates strongly and positively with catecholamine-stimulated lipolysis in adipocytes. This correlation is probably causal, because overexpression of miR-378 in human adipocytes increases catecholamine-stimulated lipolysis. Taken together, increased miR-378 expression in ALS patients could play an etiological role in degenerative cachexia-associated adipose tissue loss via effects on adipocyte lipolysis. In contrast, inhibition of miR-378 expression could attenuate stimulated lipolysis and reduce the expression of a set of genes (LIPE, PLIN1, and PNPLA2) encoding key lipolytic regulators [71]. In our study, we observed the significant decrease of miR-378 expression in all ALS patients following Lin- cells transplantation, which might have an effect on the clinical outcomes in this patient cohort. Finally, some studies have also linked the aforementioned miR-378 with inflammation processes [72,73], as generally both lymphoid- and myeloid-related cells affect inflammation in a stimulating or inhibitory manner. In this notion, it is interesting whether the results of the miR-378 expression obtained in our study after cell transplantation may provide another potential explanation for the observed significantly lower concentration of pro-inflammatory C3 molecules in the CSF of ALS patients from group I, which obtained a greater quantity of Lin- cells during the Tx procedure.

Although our study groups were too small to validate the specificity and sensitivity of the selected miRNAs as biomarkers, on the basis of our results one can conclude that one of the miRNA profiling assets in ALS is consistency of expression tendencies in the CSF and plasma. However, we cannot compare the obtained data with control group values due to the lack of a control group for the procedure in this preliminary study, which is explained below. We propose, that miRNA-155, -206, and -378 could serve as useful biomarkers of ALS response to bone marrow-derived cell therapeutic administration, but this needs further elucidation. However, for each of the analyzed miRNAs, whether the change contributes directly to disease progression or represents a compensatory/beneficial response to cell therapy remains unclear. In particular, this is a first study of miR-378 in ALS, and a vast spectrum of miR-378 targets could provide a link between various processes underlying ALS pathophysiology. In our future studies, we will aim to further investigate the emerging role of miR-378 in ALS and its potential in diagnosis, treatment, and prognosis.

### Potential Study Limitations

Overall, our study gave some interesting results, but it did not lack some drawbacks. The first is the diversity of the investigated group—patients enrolled in the study differed considerably in terms of the disease onset (17–62 years), ALS disease duration (8–108 months), and the number of available Lin- cells transplanted into each particular patient. Also, due to the characteristics of our study based on Lin- cells injection, the experiment was conducted only in ALS patients, lacking a control group. Overall, this study was not designed, nor was large enough, to determine the efficacy of slowing or stopping the progression of ALS. In addition, the clinical progression in ALS is highly variable, and the biological factors accounting for this variability are still unknown. Therefore, for proper reasoning, we divided the study cohort arbitrary into two separated groups, which included only six participants, related to their clinical outcomes, further limiting our ability to make any conclusive statements about the therapeutic efficacy of the Lin- cells transplantation. We could only observe in these small groups the changes in molecular expression of selected proteins and miRNAs. Nevertheless, we must be cautious about ascribing the cause and effect of the performed intervention to the ALS patients recruited to the study. In the absence of a control group, it remains unclear whether changes in these parameters were due to the injected cells themselves or a response of the host tissue. Additional studies are warranted to address this study limitation.

## 4. Materials and Methods

### 4.1. Patients

The study was designed as a prospective, open-label, nonrandomized clinical trial in a single center for subjects with ALS. The trial (international number: NCT02193893) was approved (approval code: KB-0012/06/10; 25 January 2010) by the Ethics Committee of the Pomeranian Medical University in Szczecin (Poland) and performed in accordance with the Declaration of Helsinki; all patients provided written informed consent. A total of 12 patients, four females and eight males, between 21 and 65 years old (49.7 ± 13.08), with sporadic ALS according to the El Escorial Revised Criteria [74] and with a survival prognosis of over 12 months on the basis of general and neurological condition were enrolled in the study. A three-month period after the enrollment was dedicated to natural history observation, during which controlled administration of riluzole (the only ALS treatment registered in Poland) was continued. Patients over 65 were excluded from the study, as it has been previously demonstrated that cell growth of expanded in vitro stem cells is strictly related to the donor’s age [75]. Patients with familial ALS, with evidence of any concurrent illness, or receiving any medications that might affect bone marrow were excluded.

### 4.2. Assessment

The clinical progression of the disease using the Norris scale was assessed at baseline, after 1, 2, 3, 5, 7, and 12 days (during hospitalization), 18 and 28 days after injection, and three and six months after injection. This scale is a rating scale for ALS and consists of two parts, the limb Norris scale (21 items to evaluate extremity function) and the Norris bulbar scale (13 items to evaluate bulbar function). Each item is rated in four ordinal categories. In addition, activities of daily living using the amyotrophic lateral sclerosis functional rating scale (ALSFRS) were assessed prior to the cell therapy, at discharge from the clinic (day 12), and during subsequent visits. The ALSFRS scale measures a patient’s level of self-sufficiency in four domains with three items in each (rated 4–0 with a maximum score = 48): bulbar function, fine motor tasks, gross motor tasks, and respiratory insufficiency. Based on the functional condition of patients three months after cell injection (assessed using the ALSFRS and Norris scale), we divided them into two groups: I—patients whose condition did not decrease based on results from at least one scale assessment (*n* = 6); and II—patients whose condition deteriorated based on results from both neurological tests (*n* = 6).

### 4.3. Cells

BM from the patients was obtained after informed consent was given. BM samples (40–50 mL) were aspirated in local anesthesia, from the posterior iliac crest of recruited patients and subsequently resuspended in a collecting medium (phosphate-buffered saline (PBS), pH 7.2) and heparin (20 U/mL; Gibco, Waltham, MA, USA). BM samples were lysed in BD PharmLyse Lysing Solution (BD Biosciences, San Jose, CA, USA) for 15 min at room temperature in the dark and washed twice in phosphate-buffered saline. The obtained suspension of BM nucleated cells (NCs) was subjected to immunomagnetic separation procedures (MiniMACS, Miltenyi Biotec, Auburn, AL, USA). Lineage-negative SPCs (7.89 ± 5.77 × 10^6^) were isolated from non-separated NCs using immunomagnetic isolation and a lineage cell depletion kit (Miltenyi Biotec, Auburn, AL, USA), as described [15], according to the GMP conditions. Before implantation, the cells were maintained in 2 mL of sterile PBS.

### 4.4. Administration Procedure

The suspension of Lin- SPCs in PBS (2 mL) was administered into the subarachnoid space by lumbar puncture between the lumbar vertebrae L3/L4 or L4/L5. The number of Lin- SPCs administered differed between the groups: group I received 11.95 ± 5.76 × 10^6^ cells, while group II received 4.53 ± 3.19 × 10^6^ cells. The cell suspension was slowly injected into the subarachnoid space for 2–3 min. Then, patients were maintained in a supine position for 24–48 h.

### 4.5. Safety Endpoints

We assessed the safety of Lin- SPCs transplantation by the development of immediate or delayed adverse events. Immediate reactions included respiratory failure, allergic reactions, local complications, systemic complications, paralysis, or sensory loss below the level of the injection site. Delayed reactions included intraspinal tumor formation or syringomyelia, persistent sensory loss, or paralysis not due to the progression of the disease.

### 4.6. Molecular Analysis

For the molecular assays, we collected peripheral blood and cerebrospinal fluid samples at different timepoints: peripheral blood was collected on the day of cell transplantation (day 0), three days, seven days, one month, three months, and six months after transplantation, while CSF was collected on day 0 and one month after the procedure. The peripheral venous blood (ca. 10 mL) was collected into tubes containing EDTA as an anticoagulant.

#### 4.6.1. Multiplex Assay

Concentrations of various factors (BDNF, NGF, CRP, C3, C4, ANGP2, and VEGF) in the CSF were quantified by multiplex fluorescent bead-based immunoassays (Luminex Corporation, Austin, TX, USA) in samples collected on the day of the cell injection and one month later. Additionally, we also assessed levels of BDNF and NGF in patients’ plasma on the day of injection, three and seven days after treatment, and three and six months after treatment. The procedure was performed according to the manufacturer’s protocol, as previously described [76].

#### 4.6.2. qRT-PCR

miRNA isolation and expression analysis was performed in the cerebrospinal fluid, and blood plasma was collected before cell administration and one month after cell transplantation. miRNA was isolated from the plasma (200 μL) and cerebrospinal fluid (200 μL) with the NucleoSpin^®^ miRNA Plasma Kit (Macherey Nagel, Düren, Germany), following the manufacturer’s protocol. Total miRNA was eluted in 30 μL nuclease-free water. Next, 25 fmol of miR-39 from C.elegans (sequence: 5′-TCACCGGGTGTAAATCAGCTTG-3′, synthetized by the Institute of Biochemistry and Biophysics, Polish Academy of Sciences) was added to each sample as a spike-in control. Next, reverse transcription was performed with 7 μL of samples using NCode miRNA First-Strand cDNA Synthesis Kit (Thermo Fisher Scientific, Waltham, MA, USA) following the manufacturer’s protocol. Subsequently, preamplification was performed as described in Jazwa et al. [77]. Quantitative assessment of the expression of the selected miRNAs (miRNA-1, miRNA-133a, miRNA-155, miRNA-206, and miRNA-378) was performed by qRT-PCR using 2 μL of 10× diluted samples obtained after preamplification, SYBR Green PCR Master Mix (SYBR Green qPCR Kit, Sigma-Aldrich, St. Louis, MO, USA), a universal reverse primer for miRNAs qPCR supplied with NCode miRNA First-Strand cDNA Synthesis Kit, and miRNA specific primers. Relative quantification of expression of selected target miRNAs was performed with the comparative Ct (cycle threshold) method. The relative quantization value of the target, normalized to spike-in control, was expressed as 2^−Δ*C*t^.

### 4.7. Statistical Analysis

GraphPad Prism software (v.6.1) was applied for all mathematical calculations, including mean and standard deviation. ANOVA with post-hoc Tukey’s test was used to calculate statistical significance; a *p*-value < 0.05 was considered significant.

## 5. Conclusions

We have shown that autologous bone marrow-derived lineage-negative early hematopoietic cells injected intrathecally are a safe and feasible source of material for transplantations to the CNS environment aimed at trophic support provision. Lin- cells intrathecal injection appeared to be a safe and well-tolerated procedure without transient or chronic adverse events. Therefore, with proper enrollment criteria and repeated cell applications, it may be possible to sustain their potentially beneficial effects, thus causing a gradual decrease or stop in ALS progression. Despite the variation in individual response arising, inter alia, due to different stages of the disease during enrollment, our preliminary results suggest that single intrathecal injection of bone marrow-derived early hematopoietic cells may cause some positive changes in various cytokines concentrations. Furthermore, our findings showed consistent miRNA expression profiles in the CSF and blood plasma, providing some presumable links between miRNA expression and the effects on the disease pathology; however, this needs further clarification. These data suggest that miR-206 or miR-133a in the CSF may also have a specific protective function at the synapse in ALS, where denervation occurs following motor neuron cell death. To our knowledge, this is the first study on miRNA-378 in ALS, which might potentially serve as a biomarker of disease progression. In our future studies, we aim to further investigate the role of miRNA-378 and its targets in the pathophysiology of ALS.

Further clinical and experimental studies need to be conducted in the future to fully define the exact molecular roles of different miRNA in the pathogenesis of different forms of ALS disease in humans. The use of miRNAs as disease biomarkers may have strong potential, not only for early diagnosis, but also for predicting disease severity and evolution and for improving phenotype classification and group stratification in the planned clinical trials.

## Figures and Tables

**Table 1:** Characteristics of both groups together with the amyotrophic lateral sclerosis functional rating scale (ALSFRS) and Norris scale results. Tx: transplantation.

Characteristic		Group I (*n* = 6)	Group II (*n* = 6)	*p*-Value
Age (mean ± SD, years)		48.7 ± 15.5	50.7 ± 10	*p* = 0.8133
Age at disease onset (mean ± SD, years)		45.5 ± 17	47.3 ± 10.4	*p* = 0.8141
Sex (male/female)		4/2	4/2	*p* = 1
Symptom duration (mean ± SD, months)		39.3 ± 27.39	37.3 ± 32.14	*p* = 0.9178
Number of Lin- cells administered (mean ± SD)		11.95 ± 5.76 × 10^6^	4.53 ± 3.19 × 10^6^	*p* = 0.0365
ALSFRS score (mean ± SD)	Before Lin- Tx	26.3 ± 2.8	15.5 ± 3.15	*p* = 0.0002
	3 months after Lin- Tx	25.5 ± 3.6	12.6 ± 1.7	*p* = 0.0002
	6 months after Lin- Tx	23 ± 6.4	10.5 ± 1.5	*p* = 0.0491
Norris scale score (mean ± SD)	Before Lin− Tx	84.3 ± 4.4	58 ± 6.4	*p* < 0.0001
	3 months after Lin- Tx	86 ± 5.9	50.3 ± 3.7	*p* < 0.0001
	6 months after Lin- Tx	81.6 ± 12.2	38 ± 6.6	*p* = 0.0007

## Supplementary Materials

Supplementary materials can be found at http://www.mdpi.com/1422-0067/19/5/1312/s1.

## Abbreviations

ALS	amyotrophic lateral sclerosis
ALSFRS	the amyotrophic lateral sclerosis functional rating scale
ANGP2	angiopoietin 2
BBB	blood-brain barrier
BDNF	brain-derived neurotrophic factor
BM	bone marrow
CNS	central nervous system
CRP	C-reactive protein
CSF	cerebrospinal fluid
GDNF	glial cell line-derived neurotrophic factor
HSC	hematopoietic stem cell
HSPC	hematopoietic stem/progenitor cell
LIN-	lineage-negative cells
NGF	nerve growth factor
NT	neurotrophin
NTF	neurotrophic factor
PBS	phosphate buffered saline
PSC	pluripotent stem cell
qRT-PCR	quantitative reverse transcription polymerase chain reaction
SC	stem cell
SPC	stem/progenitor cell
Tx	transplantation
VEGF	vascular endothelial growth factor
