# Supplementary material for: Safety and Feasibility of Lin- Cells Administration to ALS Patients: A Novel View on Humoral Factors and miRNA Profiles
Source: Int J Mol Sci. 2018 Apr 27;19(5):1312. doi: 10.3390/ijms19051312 (PMC5983708; doi:10.3390/ijms19051312)
Supplement: Supplementary file 1 [file ijms-19-01312-s001.pdf]

Supplementary data: Table 1. Results of laboratory tests performed on the obtained samples.

Patient's number	Group	Age [years]	Sex	leukocytes [x10^3/µl]	erythrocytes [mln/µl]	Hb [g/dl]	Ht [%]	PLT [x10^3/µl]	CRP [mg/l]
2	I	35	F	7,8	4,28	14,3	42,3	333	1
3	I	64	M	5,1	4,85	14,2	42,5	272	1
5	I	21	M	4,43	4,55	14,7	41,4	239	1,2
6	I	53	M	8	5,07	13,9	41	271	4,6
8	I	60	F	7,3	4,31	12,8	38,1	326	1,1
12	I	59	M	8,86	5,3	16,1	44,6	282	1
1	II	40	M	9,3	5,75	16,3	46,9	308	3,5
4	II	40	M	5,6	5,51	16,2	45,7	175	1,7
7	II	48	F	9,9	4,84	14	42,4	213	1
9	II	63	M	4,6	4,33	13	40	197	1
10	II	48	F	5,8	4,82	14,4	42,1	244	1
11	II	65	M	5,31	5,2	16,3	45,4	191	5,9

Patient's number	Group	fibrinogen [mg/dl]	Na [mmol/l]	K [mmol/l]	creatinine [mg/dl]	AST [U/l]	ALT [U/I]	CPK [U/l]
2	I	232,7	137	4,38	0,61	17	14	155
3	I	322,2	137	4,38	0,7	31	28	359
5	I	369	144	4,18	0,6	18	12	228
6	I	315,1	141	4,2	0,58	25	21	232
8	I	347,5	141	4,2	0,62	81	87	157
12	I	304,5	140	4,22	0,83	26	22	506
1	II	328,5	139	4,29	0,58	22	24	838
4	II	265,9	139	3,81	0,7	25	45	216
7	II	263,8	141	4,36	0,76	15	11	89
9	II	306,4	143	3,89	0,65	26	30	67
10	II	270,7	141	4,28	0,45	31	30	39
11	II	288,3	143	4,3	0,74	48	40	209

Hb - hemoglobin, Ht - hematocrit, PLT - platelet count, CRP – C-reactive protein, AST - aspartate aminotransferase, ALT - Alanine transaminase, CPK - creatine phosphokinase
